# Black pleural effusion due to pancreatic pseudocyst: A case report

**DOI:** 10.1097/MD.0000000000009043

**Published:** 2017-12-15

**Authors:** Feng Guo, Junli Wu, Yunpeng Peng, Min Tu, Bin Xiao, Cuncai Dai, Kuirong Jiang, Wentao Gao, Qiang Li, Jishu Wei, Jianmin Chen, Chunhua Xi, Zipeng Lu, Yi Miao

**Affiliations:** a Pancreas Center, First Affiliated Hospital; b Pancreas Institute, Nanjing Medical University, Nanjing, P.R. China.

**Keywords:** black pleural effusion, complication, pancreatic pseudocyst, surgery

## Abstract

**Rationale:**

Black pleural effusion (BPE) is an extremely uncommon type of pleural fluid, which can be due to infection, primary or metastatic malignancy, and hemorrhage. As reported in previous studies, BPE is also observed in some patients with pancreatic pseudocyst.

**Patient concerns:**

We herein reported a case of a 14-year-old female patient who was admitted to our center with a history of cough for 1 and a half months and right chest pain for 1 month. Before this, she was consecutively hospitalized in 3 different hospitals due to the same symptoms. However, the previous treatments were ineffective due to the lack of a definitive diagnosis. Laboratory examination of the pleural effusion showed BPE with a high amylase concentration. Chest x-ray and computed tomography (CT) showed massive pleural effusion, more prominent in the right chest. CT and MRCP of the abdomen showed a cystic lesion located in the tail of the pancreas, which entered the chest cavity via an esophageal hiatal hernia.

**Diagnoses:**

pancreatic pseudocyst.

**Interventions:**

After confirming that the tumor was a pancreatic pseudocyst by intraoperative biopsy, internal drainage to the jejunum was performed.

**Outcomes:**

The postoperative recovery was rapid and without complications, and the final discharge diagnosis was idiopathic pancreatic pseudocyst (without history of pancreatitis or pancreatic injuries) with BPE of the right chest.

**Lessons:**

This case demonstrates that massive BPE could present as a rare complication of pancreatic pseudocyst, and surgery is a potential treatment for such patients.

## 1 Introduction

Pleural effusion is a common clinical symptom in patients with various diseases, such as tuberculosis, intrathoracic malignancies, and hypoproteinemia.^[1]^ According to the color of the fluid, pleural effusion can be divided into yellow, reddish, and black. Yellow (serous) and reddish (blood-tinged) pleural effusions are common and well-studied, while black pleural effusion (BPE) is rare and has been investigated to a lesser degree.^[2]^ Pleural effusion requires treatment because it can result in several fatal conditions, including respiratory failure and heart failure.^[3]^ Pleural effusion can be relieved provisionally with symptomatic treatments, such as thoracentesis and drainage, but frequently relapses.^[4]^ Therefore, the key step in the treatment of pleural effusion is to diagnose and treat the underlying disease; with BPE, an underlying etiology is infrequently diagnosed.

Pancreatic pseudocyst was reported as an etiology of BPE in several studies.^[5,6]^ Pancreatic pseudocyst is a type of cystic tumor that most commonly occurs as a complication of acute or chronic pancreatitis, pancreatic injuries, and pancreatectomy.^[7,8]^ The clinicopathologic features of this cyst include a cystic wall without an epithelial cell lining and black cystic fluid with a high amylase concentration. Pancreatic pseudocyst can result in pleural effusion through different mechanisms, such as cystic fluid leakage leading to reactive effusion by stimulating the septum transversum and cystic fluid directly entering the pleural cavity to cause BPE.^[5]^ Here, we report a rare mechanism of pancreatic pseudocyst-induced BPE: massive pancreatic pseudocyst directly entering the pleural space via an esophageal hiatal hernia.

## 2 Case report

The 14-year-old female patient was admitted to our center on October 24, 2016 with chief complaint of cough for 1 and a half months and right chest pain for 1 month. The main symptoms were as follows: cough persisting for 1 and a half months without obvious causative factor; transient fever, with a peak temperature of 38°C; dull pain in the right chest beginning 1 month before presentation, aggravated with breathing and coughing; and exacerbation of pain. On physical examination, no abnormalities were observed.

**Figure 1:**
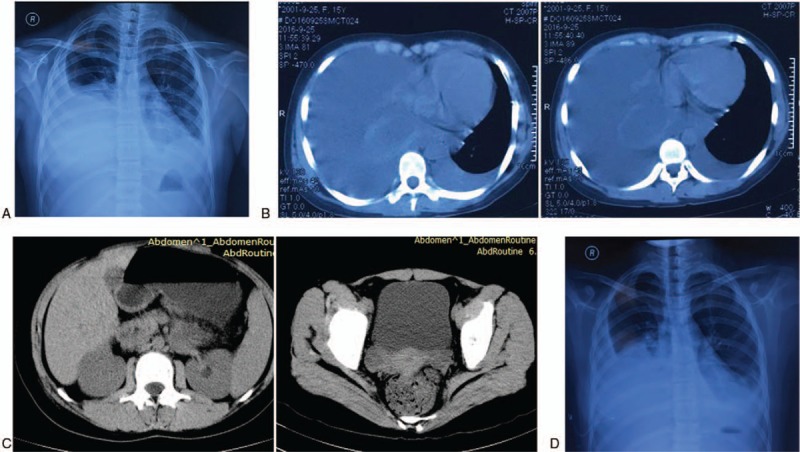
Radiographic examination at the second Affiliated Hospital of Nanjing Medical University. (A, B) X-ray and computed tomography (CT) of the chest showed massive bilateral pleural effusion, more prominent on the right side. (C) CT of the whole abdomen demonstrated a mass in the body of the pancreas and nodules in the bilateral adnexal regions. (D) Repeat chest x-ray showed finding similar to the previous x-ray (September 25, 2016).

On September 25, 2016, she visited the second Affiliated Hospital of Nanjing Medical University for cough and chest pain. To clarify the diagnosis, a series of imaging studies and blood laboratory test were performed. X-ray (September 25, 2016) and computed tomography (CT) (September 25, 2016) of the chest showed massive bilateral pleural effusion, more severe on the right side (Fig. 1A and B); CT of the abdomen demonstrated a mass (4.0 × 2.5 cm) in the body of the pancreas, and nodules (diameter, 2.0 cm) in the bilateral adnexal regions (Fig. 1C). However, gynecologic ultrasonography did not show any abnormalities in the bilateral adnexal regions. The immunological biomarker ASO was increased (310.0 IU/mL), as was the tumor biomarker CA125 (78.23 U/mL). Other results of blood laboratory tests were within the normal ranges. To investigate the pathogenesis and relieve the patient's symptoms, thoracocentesis was conducted on September 27, 2016. Pleural effusion routine testing showed the following findings: bloody, slight feculent, rivalta test (±) fluid, karyocyte count 450 × 10^6^ L^−1^, monocyte > polykaryocyte; ADA 21 U/L, chloridion 112.3 mmol/L, protein 49.5 g/L, CA125 521.80 U/mL, and CA199 52.02 U/mL. Tumor cells and mycobacterium tuberculosis were not detected in the pleural effusion. These results preliminarily excluded malignancy or tuberculosis as the cause of the pleural effusion. On September 28, repeat chest x-ray showed findings similar to the previous x-ray findings (Fig. 1D), without improvement. To further clarify the diagnosis, the patient was transferred to Nanjing Chest Hospital.

**Figure 2:**
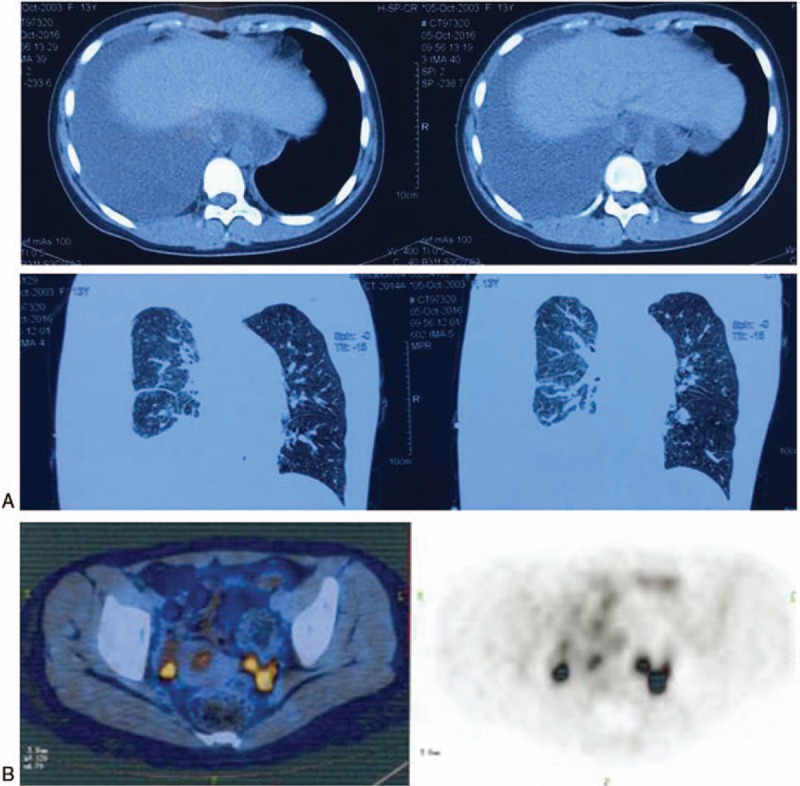
Radiographic examination at Nanjing Chest Hospital. (A) Computed tomography (CT) of the chest showed massive fluid in the right-sided chest. (B) Whole body PET-CT showed masses located in the bilateral adnexal regions.

She was admitted to Nanjing Chest Hospital on September 29, 2016. Laboratory testing of blood and pleural effusion were performed. The positive results were as follows: immunological test (blood, September 30, 2016): tubercle bacillus antibodies (+), P-ANCA (+), anti-MP IgM (+); tumor markers (blood, September 30, 2016): CA125 118.00 U/mL; pleural effusion routine testing (September 30, 2016): old hematodes exudate. Ultrasound of the chest showed bilateral pleural effusions, and ultrasound of the abdomen showed no significant findings. After symptomatic treatment, repeat chest CT was performed (Fig. 2A), showing findings suggestive of massive fluid in the right chest. To evaluate whether the BPE was due to malignancy, whole body PET-CT was performed. Results showed possible malignancies located in the bilateral adnexal regions (Fig. 2B). Considering the increase in CA125 level, the BPE was thought to potentially be due to metastatic gynecologic malignancy. To confirm this hypothesis, the patient was transferred to the pediatric department of the first Affiliated Hospital of Nanjing Medical University.

**Figure 3:**
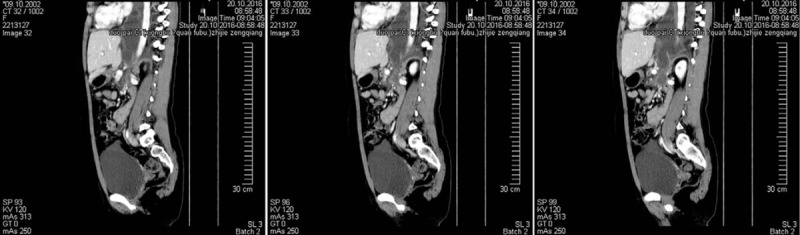
Radiographic examination in the pediatric department of our Hospital. Computed tomography (CT) of the chest and abdomen suggested massive fluid in the bilateral pleural cavities (right greater than left), and a cystic lesion possibility emanating from the tail of the pancreas entering the chest via an esophageal hiatal hernia.

On October 10, 2016, she was admitted to the pediatric department of our Hospital. The possibility of gynecologic malignancy was preliminarily excluded based on the recommendation of a gynecologist, and the masses in the bilateral adnexal regions were considered to be corpus luteum. Repeat CT of chest and abdomen showed massive fluid in the bilateral pleural cavities (right greater than left), and a cystic lesion possibly extending from the tail of the pancreas to the chest via an esophageal hiatal hernia. However, laboratory blood tests did not demonstrate any significant abnormalities (Fig. 3). Thoracocentesis was performed to clarify the diagnosis and relieve the patient's symptoms on October 17, 2016. Unfortunately, the results of laboratory tests based on pleural effusion were still not suggestive of an underlying diagnosis, and her symptoms did not improve. To evaluate the association between the pancreatic cystic lesion and pleural effusion, the patient was transferred to our center.

**Figure 4:**
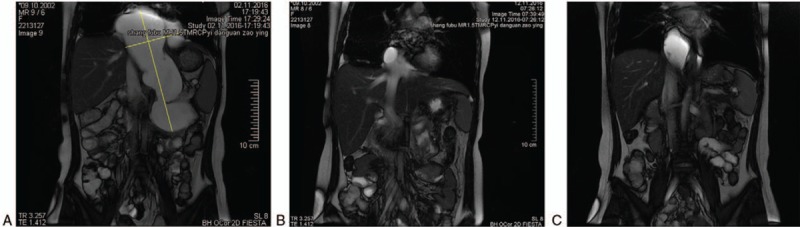
Radiographic examination at our center. (A) Preoperative MPCP revealed that the cystic lesion in the pancreas and the chest communicated via an esophageal hiatal hernia. (B, C) Postoperative MPCP showed reduced pleural effusion and a small cystic lesion in the right chest.

After admission to our center, we first performed thoracocentesis for symptomatic relief and to assist in diagnosis. Amylase was detected in the pleural effusion, and the results suggested that the amylase level was significantly increased. The increased concentration of amylase indicated that the cystic lesion in the pancreas and the chest were in communication, and the BPE might be due to leakage of the cystic lesion. To confirm this diagnosis, we performed MPCP test (Fig. 4A); the results revealed that the cystic lesion in the pancreas and the chest were interlinked via an esophageal hiatus. We performed exploratory laparotomy on November 03, 2016. A huge cystic lesion was located in the left upper abdomen. The color of the cystic fluid was similar to that of the pleural effusion. Intraoperative biopsy showed that the wall of the cystic lesion was not covered with epithelial cells, and the cyst was diagnosed as pseudocyst. Therefore, internal drainage to the jejunum was performed, and the operation was completed without complications. Laboratory tests of cyst fluid showed the following: amylase 22634.00 U/L; bacterial culture negative; tumor markers: CEA 9.8 ng/mL, CA199 > 1000 U/mL, CYFRA21-1 76.4 ng/mL, NSE 25.0 ng/Ml, and CA125 53.2 U/mL.

The patient's postoperative recovery was generally satisfactory, and she was discharged from the hospital on November 11, 2016. The final diagnosis was idiopathic pancreatic pseudocyst with BPE of the right chest. Repeat MRCP on November 12 and December 19 demonstrated a small pleural effusion and small cystic lesion in the right chest (Fig. 4B and C).

## 3 Discussion

The morbidity of pancreatic pseudocyst rupture-induced BPE is extremely low and diagnostic and therapeutic strategies are undefined due to the poor understanding of this disease. In some cases, patients with pancreatic pseudocyst present to the hospital with BPE as the only symptom. In these cases, the main diagnostic goal is to clarify the characters of the pleural effusion, especially the presence of an increased concentration of amylase (>1000 IU/L).^[9]^ The presence of a pseudocyst in the pancreas or even breaks into the chest is also necessary. Furthermore, some studies suggest that the significantly higher ratio of pleural total bilirubin and serum total bilirubin is also supportive of the diagnosis.^[10–12]^

For the treatment of this disease, available methods include the following: pancreatic rest, including NJ feeding, chest drainage, somatostatin analogue treatment, and pancreatic enzyme replacement therapy; endoscopic therapy, such as endoscopic retrograde cholangiopancreatography; and surgical treatment.^[13,14]^ Furthermore, the management of primary diseases resulting in pancreatic pseudocyst, such as pancreatitis and pancreatic injuries, is equally important. However, our patient did not have a history of any diseases that would result in pancreatic pseudocyst. Although we appropriately treated the pancreatic pseudocyst with internal drainage, it is likely to recur because of the persistent presence of an, as yet, unidentified precipitating factor. Therefore, this patient should be frequently followed up to detect early recurrence.

The CA125 level in this patient was increased. CA125 is a tumor marker commonly identified in ovarian cancer; it can also be increased in other non-ovarian malignancies, such as breast cancer, gastric cancer, pancreatic cancer, and lung cancer.^[15]^ Furthermore, CA125 may also be increased in some non-neoplastic diseases, such as pancreatitis.^[16]^ Therefore, elevated CA125 level in this patients might be associated with the presence of pancreatic inflammation. Although the possibility of adnexal lesions was preliminarily excluded based on consultation with a gynecologist, we also evaluated the adnexal lesions by following CA125 level and MRI after surgery. The blood level of CA125 (December 16, 2016) was 12.23 U/mL, and MRI (December 19, 2016) did not demonstrate adnexal lesions.

All recommendations above are based on personal experience and literature reviews. Therefore, well-designed studies with large sample sizes are needed to investigate the nature of this disease and determine the most appropriate diagnostic and treatment strategies.
